# MLN-8237: A dual inhibitor of aurora A and B in soft tissue sarcomas

**DOI:** 10.18632/oncotarget.7335

**Published:** 2016-02-12

**Authors:** Jayasree S. Nair, Gary K. Schwartz

**Affiliations:** ^1^ Jennifer Goodman Linn Laboratory of New Drug Development, Department of Medicine, Memorial Sloan-Kettering Cancer Center, New York, New York, USA

**Keywords:** apoptosis, polyploidy, Aurora A, Aurora B, sarcoma

## Abstract

Aurora kinases have become an attractive target in cancer therapy due to their deregulated expression in human tumors. Liposarcoma, a type of soft tissue sarcoma in adults, account for approximately 20% of all adult soft tissue sarcomas. There are no effective chemotherapies for majority of these tumors. Efforts made to define the molecular basis of liposarcomas lead to the finding that besides the amplifications of CDK4 and MDM2, Aurora Kinase A, also was shown to be overexpressed. Based on these as well as mathematic modeling, we have carried out a successful preclinical study using CDK4 and IGF1R inhibitors in liposarcoma. MLN8237 has been shown to be a potent and selective inhibitor of Aurora A. MLN-8237, as per our results, induces a differential inhibition of Aurora A and B in a dose dependent manner. At a low nanomolar dose, cellular effects such as induction of phospho-Histone H3 (Ser10) mimicked as that of the inhibition of Aurora kinase A followed by apoptosis. However, micromolar dose of MLN-8237 induced polyploidy, a hallmark effect of Aurora B inhibition. The dose dependent selectivity of inhibition was further confirmed by using siRNA specific inhibition of Aurora A and B. This was further tested by time lapse microscopy of GFP-H2B labelled cells treated with MLN-8237. LS141 xenograft model at a dose of 30 mg/kg also showed efficient growth suppression by selective inhibition of Aurora Kinase A. Based on our data, a dose that can target only Aurora A will be more beneficial in tumor suppression.

## INTRODUCTION

Mitosis, despite being the shortest phase of the cell-cycle, orchestrates major changes in multiple cellular components in actively proliferating cells, resulting in the division of duplicated sets of chromosomes and two genetically identical daughter cells. Failure of cell-cycle checkpoint regulations often results in aneuploidy and genetic instability, leads in cancer [1]. Defects in mitotic signaling pathways, might lead to unrestrained growth, one of the hallmarks of cancer cells. The significance of mitotic machinery as a validated drug target has been proved using taxanes and Vinca alkaloids effectively in the treatment of many tumor types [2, 3]. Other targets for anticancer drug development proposed later on against the mitosis-regulating molecules include the aurora kinases [4], polo-like kinases [5] and the cyclin-dependent kinases [6].

The Aurora kinases are a family of serine/threonine kinases comprises Aurora A, B and C, with a highly conserved C-terminal kinase domain. They play critical role in G2 and M phases of the cell-cycle. Their divergent localization and function are determined by the separate protein complexes they are associated with. Aurora A and B cooperate closely in regulating chromosome assembly and alignment, metaphase spindle stability and anaphase MT dynamics [7]. Aurora A is critical in the maintenance of genomic integrity because its role includes centrosome assembly, maturation and proper functioning of the mitotic spindle apparatus [8, 9]. The role of Aurora B is mainly in the mitotic spindle checkpoint complex, histone-H3 phosphorylation, and cytokinesis [10–13]. Both overexpression and gene amplification of Aurora A have been characterized in human tumors and have been shown to correlate with tumor proliferation rates and prognostic markers [14–18]. Overexpression of Aurora B is also an established characteristic of certain human cancers [19–21].

Due to the critical role for Aurora kinases A and B in mitosis as well as their deregulated expression in tumors, Aurora kinases became one of the most important antitumor targets. Growth suppression upon inhibition of Aurora kinases using RNA interference as well as other methods led to the development of several small molecule inhibitors. ZM447439, AZD 1152, MK-0457, MK8745, PHA739358, MLN8054 and MLN8237 are few of the small molecule inhibitors of Aurora Kinase B, A or A/B which are under different stages of clinical development [4, 22–28, 45]. Our studies on AZD1152 have shown its selective inhibition of Aurora B kinase and the effect of endoreduplication and polyploidy at a very low nanomolar concentration despite the p53 status [23]. However, MK-8745, a selective Aurora A Kinase inhibitor was shown to induce apoptosis versus polyploidy in a p53 dependent manner [25].

Though a rare cancer in adults, sarcoma constitutes approximately 15% of all childhood cancers with a special prevalence of bone sarcomas including Ewing's and osteosarcoma. In adults there are at least 50 different soft tissue sarcoma subtypes that have unique molecular and biological phenotype. Efforts have been made to define the molecular basis of each of these tumors. For example liposarcoma which is the most common type of soft tissue sarcoma in adults, accounting for approximately 20% of all adult soft tissue sarcomas, is characterized by amplifications of CDK4 and MDM2 [29]. Aurora Kinase A has also been shown to be overexpressed in patients with this disease. It has been shown in MPNST that *HMMR*/RHAMM is critical in sensitizing cells to MLN8237 [42]. Though, Aurora Kinase inhibitors in combination with polo like kinase inhibitor shown to add efficacy in nasopharyngeal carcinoma [43], our studies did not show any added effect when combined with polo like kinase inhibitor. Effect of MLN-8237 on single cell analyses has shown a complex cellular response and its dose dependent effect [45]. For the vast majority of these tumors there are no effective chemotherapies. Therefore, by exploring the molecular basis of these tumors, it is hoped that we can identify “druggable” targets that can be translated into cancer medicine.

In the present study, we evaluated the biological activity and effect of MLN-8237, a putative Aurora A inhibitor against a panel of sarcoma cell lines.

Our results indicate that the drug inhibits growth at low nanomolar concentrations by inhibiting Aurora A with induction of apoptosis and at higher micromolar concentrations it inhibits growth by inhibiting Aurora B with induction of polyploidy.

## RESULTS

### MLN-8237 induces growth suppression in Sarcoma

**Figure 1:**
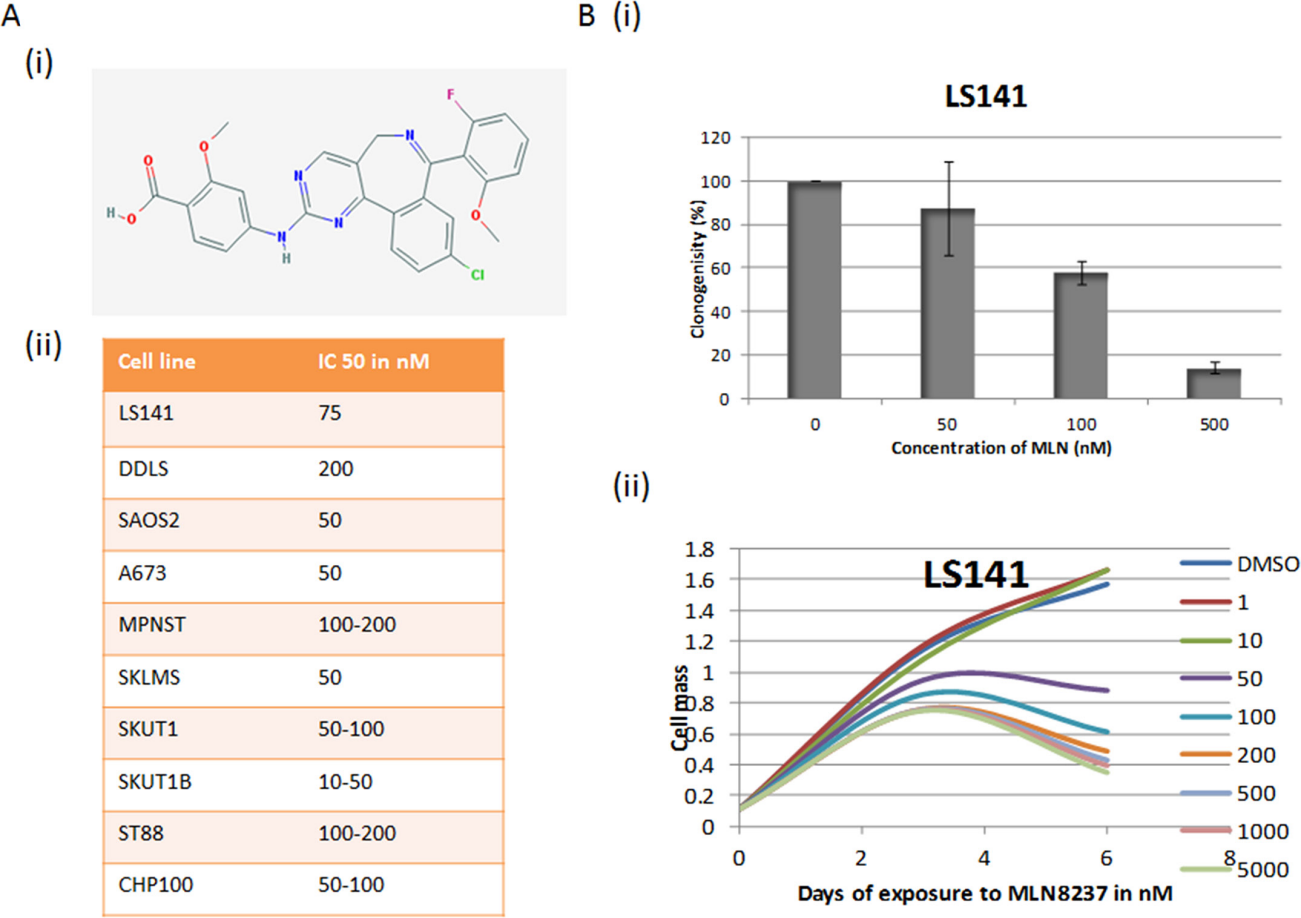
Aurora Kinase Inhibitor MLN8237 induces growth suppression (**A**) (i) Chemical structure of MLN-8237. (ii) IC-50 values of MLN-8237 in Sarcoma cell lines. IC-50s were determined by cell proliferation assay by using the Dojindo Cell Counting Kit done in six replicates. (**B**) (i) Dose curve for LS141 cells by clonogenic assay (averages of triplicates). (ii) Time-dose growth curve of LS141 as determined by colorimetric proliferation assay (average of six duplicates).

Sarcoma cell lines were tested for their sensitivity towards MLN 8237, a selective inhibitor of Aurora A kinase. Chemical structure of the small molecule inhibitor, MLN 8237 is shown in Figure 1A(i) and (ii) summarizes the IC50 values obtained for different sarcoma cell types as determined by colorimetric cell proliferation assay. MLN-8237 seems to be very potent inhibitor of tumor growth across multiple sarcoma subtypes. *In view* of the reported overexpression of Aurora A in liposarcoma, our initial focus was to assess the effect of MLN-8237 in liposarcoma cells. As a first step we evaluated the time-dose growth curve for LS141 by clonogenic and colorimetric proliferation. As shown in Figure 1B(i) the ability of a single cell to form colony is inhibited almost completely at 500 nM with an IC50 of around 100 nM. By the colorimetric assay, the percentage of proliferating cells decreased to about 30% with 100 nM with an IC50 of 50 nM.

### MLN-8237 inhibits aurora A kinase at lower and aurora B kinase at higher concentrations

**Figure 2:**
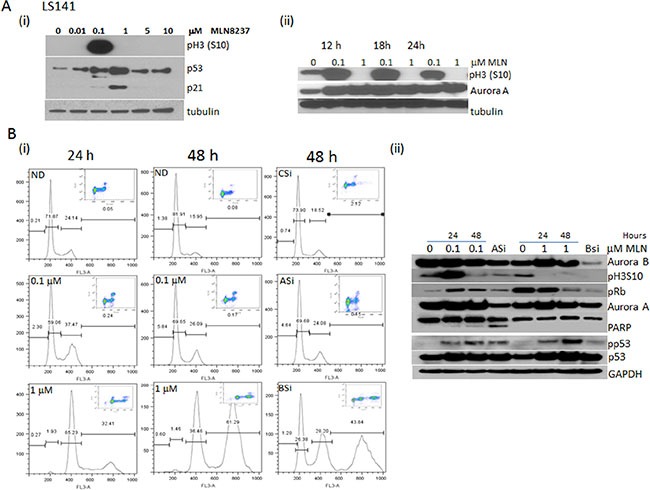
Dose dependent differential inhibition of MLN-8237 recapitulates the effect of Aurora A and Aurora B knockdown (**A**) (i) LS141 cells were exposed to increasing doses (10–10000 nmol/L) of MLN-8237 for 24 h and the phospho Histone H3 (Ser10), p53 and p21 were determined by Western blot analysis. (ii) Time dependent induction of phospho Histone H3 (Ser10) upon exposure to 100 and 1000 nM MLN-8237 by Western blot analysis. (**B**) (i) Flow cytometric analysis of LS141 cells probed for phospho-MPM2 followed by propidium iodide showing mitotic accumulation and cell cycle distribution after treatment with 0.1 or 1 mmol/L MLN-8237 for 24 and 48 h or transfected with control siRNA (CSi) or siRNA specific for Aurora A (ASi) or Aurora B (BSi) for 48 hours. (ii) Western blot analysis of LS141 cells exposed to 0.1 and 1 μM MLN-8237 for 24 and 48 hours along with Aurora A and Aurora B inhibition by specific siRNA probed for pH3 (S10), pRb, cleaved PARP, p53, p21, Aurora A, Aurora B and GAPDH as loading control.

Time-dose and concentration dependency of MLN-8237 to inhibit Aurora A and B was tested in LS141 by choosing a range of concentrations from 10 nM to 10 μM from 12 to 48 hours of exposure. We elected to monitor phospho histone H3 (Ser10) as this accumulates in the setting of the mitotic arrest induced by Aurora A inhibition but is in itself a substrate of Aurora B such that phospho histone H3 is inhibited in the setting of Aurora B inhibition. As shown in Figure 2A(i) induction of phospho histone H3 (ser10) after 24 hours of drug exposure occurs only at a concentration of 100 nM and not at higher or even lower doses consistent with a dose dependent Aurora A effect. Also, there was an induction of p53 and p21 in a dose dependent manner. The effect on phospho Histone H3 was further confirmed and the timing for its induction was further tested by exposing LS141 cells to 100 nM and 1000 nM of MLN-8237 over a period of 24 hours. As shown in Figure 2A(ii), with 100 nM of MLN-8237 the induction of phospho histone H3 (Ser10) occurred at approximately 12 hours, peaks at 18 hours, then starts to decline at 24 hours. It is interesting to note that at 1000 nM phospho H3S10 is completely inhibited at all time points tested. At the same time, there is induction of Aurora A protein levels at both the low and high dose conditions indicating Mitotic accumulation at both concentrations (phospho MPM2 by FACScan analysis). This clearly suggests Aurora A inhibition at 100 nM (high phospho H3S10) and Aurora B inhibition at 1000 nM dose (ablation of phospho H3S10).

The specificity of MLN-8237 in inhibiting Aurora A at low and Aurora B at high concentrations was validated further by using specific siRNA to inhibit Aurora A and B. We have previously reported that Aurora B inhibition induced polyploidy which is distinct from Aurora A inhibition which is associated with an increase in mitotic index and apoptosis. Results from FACScan analysis is shown in Figure 2B(i). The cellular effect of mitotic arrest and apoptosis with 100 nM MLN-8237 for 48 hours is comparable to the effects upon specifically inhibiting Aurora A by using siRNA. With MLN-8237 there was an increase in mitotic fraction from 2% to 12% when compared to untreated controls at 24 hours and this was maintained though at reduced numbers for 48 hours of continuous drug exposure. The effects at 48 hours were very similar to the siRNA. Under these conditions there was also induction of a small sub-G1 peak consistent with apoptosis. In contrast, polyploidy with induction of an 8N peak was the cellular fate upon inhibiting Aurora B by specific siRNA and with 48 hours of exposure to 1000 nM of MLN-8237. The knock down of Aurora A and B as well as the apoptotic and polyploidy effects were further confirmed by western blot analysis. As shown in Figure 2B(ii) at 100 nM phospho H3 (S10) was induced followed by apoptosis (cleaved PARP) at 48 hours comparable to inhibition of Aurora A by siRNA knock down. However, only with 1000 nM of MLN-8237 and with Aurora B siRNA knockdown resulted in polyploidy with induced hypophosphorylation of Rb. As we have reported previously this is consistent with inhibition of Aurora B kinase and the corresponding induction of polyploidy. Under both conditions of Aurora A and B inhibition there was p53 and p21 induction.

### The differential effect of MLN-8237 in other cell types

**Figure 3:**
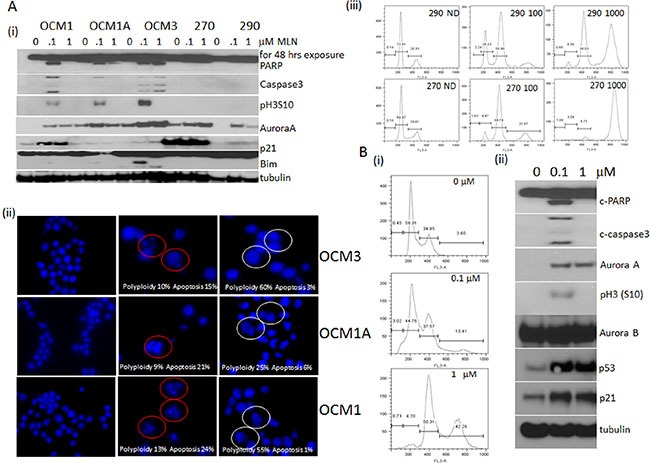
Differential inhibition of MLN-8237 in cell lines other than sarcoma (**A**) Uveal melanoma cells; OCM1, OCM1A, OCM3, Mel270 and Mel290 were exposed to 0.1 and 1 μM of MLN-8237 for 48 hours and harvested for (i) western blot analysis probing for cleaved PARP, cleaved caspase 3, phospho histone H3 (Ser10), Aurora A, p21, Bim and tubulin as loading control. (ii) Fluorescent microscopic images of DAPI stained OCM3, OCM1A and OCM1 treated with 0.1 and 1 μM of MLN-8237 for 48 hours. Normal untreated (ND) (first panel), apoptotic cells (red circles, middle panel) and polyploid cells (white circles, last panel) are marked. (iii) Mel270 and Mel290 were treated with 0, 0.1 or 1 μM MLN-8237 for 48 hours were harvested for FACScan analysis after staining with propidium iodide showing cell cycle distribution. (**B**) HCT116 cells treated with 0, 0.1 or 1 μM MLN-8237 for 48 hours were harvested for (i) FACScan analysis after staining with propidium iodide showing cell cycle distribution showing < 2N for 0.1 and 8N for 1 μM exposure. (ii) Western blot analysis of the same treatment probed for cleaved PARP, Aurora A, pH3 (S10), Aurora B, p53, p21 and tubulin as loading control.

In order to test the differential effect of MLN-8237 in other cell types, uveal cell lines were tested. Figure 3A(i) further confirms the concentration based differential effect of MLN-8237 to be true in uveal cell lines as well. Of the 5 cell lines three undergo apoptosis with induced phospho H3 (S10) at a lower concentration and lacking the apoptotic effect at a higher concentration except for OCM3. This could be due to the difference in the doubling time of the cell line. Also, the two cell lines which do not show any induction of phospho H3 (S10) had no apoptotic effect at 100 nM, though induction of Aurora A is comparable to the other cell lines. Induction of cleaved Bim was observed under apoptotic condition. Figure 3A(ii) is a comparison of the nuclei (DAPI stained) of an untreated versus treated with low or high dose of MLN-8237. As shown, there are more decondensed apoptotic bodies in the low dose (15%, 21% and 24%) and enlarged polyploid nuclei in the high dose (60%, 25% and 55%) exposed cells compared to untreated nuclei. FACScan analysis of the cells which do not undergo apoptosis showed continued proliferation with endoreduplication with an increased 8N peak (Figure 3A(iii)).

The observation was further tested in HCT116 colon carcinoma cell line and results are shown in Figure 3B and 3B(i) explains the FACScan analysis with < 2N subG1 peak, a measure of apoptotic cells and < 4N peak, a measure of polyploid cells in the low and high dose exposed cells respectively. 3B (ii) is a western blot showing induced cleaved PARP and caspase 3, a markers of apoptosis along with induced phospho H3 (S10), p53, p21 and Aurora A with low dose and a similar pattern of other protein induction except for the apoptosis and phospho H3 (S10) in the high dose.

### The concentration dependent effect of MLN-8237 using time lapse video microscopy

As we have previously shown that Aurora B inhibition induces polyploidy in HCT116 cells we further tested the effects of MLN-8237 at both high and low concentrations in this cell line. As shown in Figure 3B with 100 nM of MLN-8237 there was a slight induction of a sub G1 peak consistent with apoptosis and a marked increase in the G2M fraction with some induction of polyploidy (8N). This is in clear contrast to the 1000 nM concentration where there is no apoptotic fraction but a dramatic shift to the induction of polyploidy. By western blot analysis the low dose of MLN-8237 induced cleaved PARP and caspase 3 along with induction of phospho H3 (S0), p53, p21 and Aurora A consistent with an Aurora A effect whereas with the higher concentration though there was a similar pattern for the other proteins there was no apoptosis and inhibition of phospho H3 (S10), consistent with an Aurora B effect.

**Figure 4:**
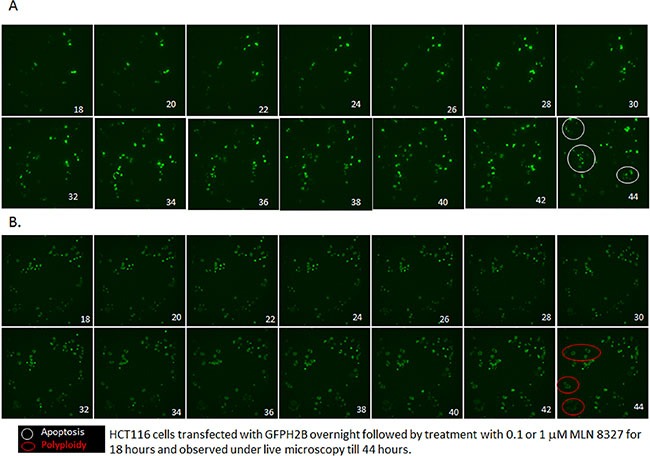
MLN-8237 induces differential effect in a dose dependent manner HCT116 cells transfected with GFP-histone 2B for 24 h were treated with 0.1 (**A**) or 1 μM (**B**) MLN-8237 and live cell imaging was done after 18 h of exposure as described in Materials and Methods. Shown are selected still images from time-lapse movies; the fate of individual cells was tracked over time. (A) Cells exposed to 0.1 μM MLN-8237 enter mitosis; stays many hours followed by apoptosis (in white circle, 30%) (Supplementary Video 1A data). (B) Cells exposed to 1 μM MLN-8237 enter mitosis after a short delay undergoes endoreduplication results in polyploidy (in red circle, 60%) (Supplementary Video 1B data).

The effect of MLN-8237 was further confirmed by time lapse video microscopy using HCT 116 cells transiently transfected with GFP H2B followed by treatment with either 1000 nM or 100 nM of MLN-8237 (Supplementary 1A and 1B, Movies). Figure 4A and 4B shows clips from the movie taken post 18 hours of exposure to MLN-8237 showing delay in mitosis, inhibition of cytokinesis and polyploidy (enlarged nuclei) at higher concentration and a delay in mitosis followed by exit and apoptosis at lower dose MLN-8237 treatment. In essence, A shows delay in mitosis followed by cytokinesis and simultaneous apoptosis (30%) and B shows a delay in mitosis followed by endoreduplication resulting in enlarged polyploid nuclei (90%). This further confirms the finding that cells upon exposure to low dose MLN-8237 undergo apoptosis following Aurora A inhibition; whereas cells exposed to high dose undergo endoreduplication and polyploidy by inhibition of Aurora B.

### MLN-8237 efficiently suppresses liposarcoma tumor growth *in vivo*

**Figure 5:**
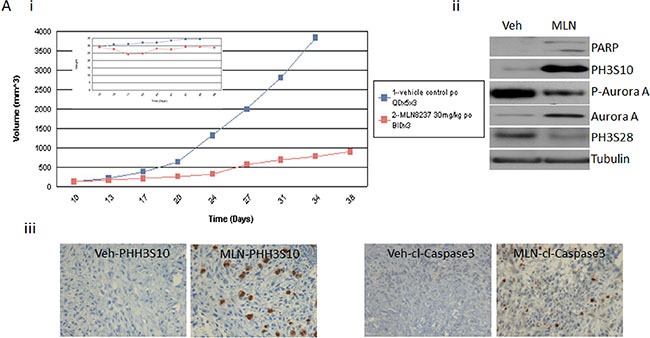
MLN-8237 induces tumor suppression in LS141 tumor *in vivo* Athymic mice were implanted with LS141 tumors and mice (*n* = 7) were treated on days 7, 14, and 21 with MLN-8237 (30 mg/kg) or vehicle as described in Materials and Methods. (**A**) (i) tumor volume was measured every 2 to 3 d, and mean tumor volume was plotted against time in days. (ii) 24 h after the final treatment, tumors were excised and analyzed by western blot analysis probing for cleaved PARP, phospho histone H3 (ser10), phospho histone H3 (ser28), Aurora A and tubulin as loading control. (iii) 24 h after the final treatment, tumors were excised and analyzed by immunohistochemistry for phospho histine H3 (ser10) and cleaved caspase 3. A representative experiment is shown.

In order to determine whether the dose dependent differential inhibition has an impact *in vivo* we tested MLN-8237 in LS141 xenografts and checked the inhibition of the pathway. Figure 5A clearly shows efficient tumor growth suppression at 30 mg/kg of MLN-8237. Unfortunately, we could not escalate the dose to study the effect of any higher doses due to toxicity. Upon analyzing the lysates from the MLN-8237 treated tumor versus the vehicle treated controls by western blot analysis, we observed induction of phospho H3 (S10) and cleaved PARP indicative of inhibition of Aurora A but not Aurora B at the dose tested (Figure 5A(ii)). Besides phospho H3 (S10), phospho (S28), an Aurora A substrate, was also tested and interestingly it was down regulated in the treated tumors further confirming Aurora A inhibition can result in inhibition of tumor growth. Immunohistochemistry of the xenografts confirmed the induction of apoptosis and phospho H3 (S10) again supporting the fact that the tumor suppression observed with MLN-8237 is predominantly a result of Aurora A (and not Aurora B) inhibition.

## DISCUSSION

Anti-mitotic agents such as taxanes and Vinca alkaloids have been clinically validated in sarcoma but the benefits of these drugs are limited, both by low levels of clinical activity, as well as cumulative toxicities such as neuropathy. The first published association of Aurora kinases with human malignancy was in 1997 when Aurora A was reported to be overexpressed in human breast cancer cells [30]. Overexpression of Aurora A and Aurora B were reported in colon, pancreatic, ovarian, bladder, liver and gastric cancers [31–34]. Later on Aurora A became established as a bona fide oncogene. MLN-8237 is an Aurora Kinase inhibitor being developed by Millennium Pharmaceuticals. MLN-8237 is an adenosine triphosphate (ATP) competitive and reversible inhibitor of Aurora Kinase A with an inhibition constant of 0.43 nM [35]. MLN-8237 showed about 200 fold more selectivity for Aurora kinase A compared to Aurora kinase B in HCT116 cells and *in vitro* kinase assays have shown 250 fold selectivity for Aurora A compared to other kinases tested *in vitro*. MLN8237 has been shown to be potent in tumor suppression in peripheral T Cell Lymphomas [46]. This agent has completed phase I trials and the dose limiting toxicity is neutropenia with a recommended phase II dose of 50 mg orally twice a day for 7 days out of a 21 days cycle. Several phase II clinical trials have been completed including a phase II clinical trial for patients with advanced sarcomas (NCI protocol #12–194, 47).

Liposarcomas are rare connective tissue cancers, account for about 18% of all soft tissue sarcomas. Of the four types of liposarcomas, well and dedifferentiated liposarcoma represent the most common biologic group of liposarcoma. Progression of WDLS to DDLS is associated with the acquisition of metastatic potential and poor patient prognosis [36]. Patients with dedifferentiated histology are at high risk for recurrence and surgical outcomes are poor for patients with rapidly growing tumors. Besides surgery, two other options are radiation and chemotherapy which also have low response in WDLS and DDLS. Anti-mitotic agents such as taxanes and Vinca alkaloids have been clinically validated in sarcoma but the benefits if this drugs are limited, both by low levels of clinical activity as well as cumulative toxicities such as neuropathy. Reports on the amplification of chromosome 12q13–15, containing oncogenes MDM2, HMGA2 and CDK4 in about 90% of well and dedifferentiated liposarcoma [37, 38] led to the development of new targeted agents. Novel targeted agents against MDM2 and CDK4 have shown promising results in preclinical studies and which are being currently tested in clinic. Z1C1, TOP2A, AURKA and IGF-1R have also been identified as targets using cell line and tissue microarray and genomic analyses [39]. Based on this study as well as mathematical modeling we have carried out a preclinical study successfully by using CDK4 and IGF1R inhibitors in liposarcoma [40].

Our results indicate that MLN-8237 is highly active against multiple sarcoma subtypes in low nanomolar concentrations even in the absence of Aurora A amplification. Though described as an Aurora A inhibitor, our results indicate that MLN8237 in fact, inhibits both Aurora A and B in a dose dependent nature. The concentration dependent induction of polyploidy by MLN8237 has also been reported in bladder cancer cell lines [41]. Interestingly, this effect was attributed to Aurora A inhibition despite the fact that polyploidy is a phenotype associated with inhibition of Aurora B and not Aurora A. Our data strongly indicate an Aurora B effect at these high (micromolar) doses. This differential effect of MLN-8237 on Aurora A and B has also been reported in Peripheral T-Cell Lymphoma cells. Here *ex vivo* treatment of PTCL derived patient cells treated with MLN-8237 induced polyploidy at high concentration consistent with an Aurora B effect. The relevance of this to the evaluation of MLN-8237 cannot be minimized as in the clinic this drug can achieve pharmacological levels of > 500 nM. A phase II clinical trial was conducted based on our results in which 72 patients with advanced and metastatic sarcoma, included 12 patients with liposarcoma. The median progression free survival in weeks was 13 (6.29–37.14) for the liposarcoma cohort [47]. This outcome exceeded the minimum bar and hence this drug worthy of further evaluation in this particular disease.

## MATERIALS AND METHODS

### Cell culture

HCT116 (colon cancer cell line) and Sarcoma cell lines; A673 and CHP100 (Ewing), MPNST and ST88 (malignant peripheral nerve sheath), LS141 and DDLS (dedifferentiated liposarcoma), SK-UT1, SK-UT1B and SK-LMS (uterine leiomyosarcoma) and SaOs2 (osteosarcoma) were maintained in RPMI with 50 U/ml each of penicillin and streptomycin, and 10% heat-inactivated fetal bovine serum, and incubated at 37°C in 5% CO_2_.

### Drugs

MLN8237, a small molecule inhibitor of Aurora A Kinase), was provided by Millennium.

### Colorimetric cell proliferation assay

The assay was done with minor changes from the manufacturer's protocol (Dojindo Molecular Technologies, Inc.) as follows. Briefly, 1,000–2,000 cells were plated in 100 μL volume per well of a 96-well plate, SiRNA transfections were done at the time of plating and treatments were done 24 h after plating. After the incubation period of 3 days with MLN8237 (0–1000 nM), the media was replaced with MEM containing 10% serum and 10% CCK-8 solution, which were further incubated at 37°C for 1 to 4 h. In this assay the amount of formazan dye generated by the activity of dehydrogenases in the cells is quantified and which is directly proportional to the number of living cells. Then the optical density at 450 nm to determine the cell viability was measured using Spectra Max 340 PC (Molecular Devices Corp).

### Clonogenic cell proliferation assay

Cells were plated, in triplicate, in 10-cm dishes at 1,000 per dish and were treated after 24 hours with indicated concentrations of MLN8237 for 24 hours. After 24 hours cells were cultured in drug-free medium for 10 to 14 days. The resulting colonies were scored after staining with 0.01% crystal violet.

### Flow cytometry

Cells were washed and fixed in 75% ice-cold ethanol before staining with propidium iodide (50 μg/mL) containing RNase (5 μg/mL) for the measurement of DNA content. Mitotic population was measured by labeling the fixed cells with phospho MPM-2 monoclonal antibody (Millipore), which recognizes specific phosphorylated epitopes present only in mitosis followed by Alexa Flour 488 antimouse secondary antibody (Invitrogen, Oregon, USA). Cells were then stained with propidium iodide containing RNase. Samples were analyzed on a FACScan (Becton Dickinson) for cell cycle distribution and mitotic index (fraction of cells positive for phospho MPM-2) using the Cell Quest software. 10,000 events were examined per sample.

### siRNA transfection

Cells were plated on 60-mm plates, and transfections using lipofectamine RNAiMAX (Invitrogen) were performed according to the manufacturer's protocol. The siRNA sequences for Aurora A and Aurora B were purchased from Dharmacon (Pittsburgh PA, 15275 United States) and Ambion Inc. (Life Technologies, 3175 Staley Road, Grand Island, NY14072, USA). Cells were harvested 48 hours after transfection for Western Blot analysis or FACScan analysis.

### Cell lysate extraction and immunoblotting

Both floating and adherent cells in whole-cell lysis buffer (50 mmol/L Tris, pH 7.4, 150 mmol/L NaCl, 1% NP-40, 1 mmol/L EDTA, 0.25% sodium deoxy cholate, 0.1 mmol/L Na_3_VO_4_, with protease inhibitor cocktail tablet (Roche)), was allowed to lyse on ice for 10 min, syringed and cleared by centrifugation in a microcentrifuge at 13,000 rpm for 10 min at 4°C. 30 micrograms of protein were fractionated by SDS-PAGE and transferred onto Immobilon membranes (Millipore). Membranes were blocked with 5% nonfat milk, probed with primary antibodies followed by incubation in horseradish peroxidase-conjugated secondary antibodies and visualized by enhanced chemiluminescence reagent (both from GE Healthcare UK Limited). The following antibodies were used in this study: mouse monoclonal to cleaved poly (ADP-ribose) polymerase (PARP), rabbit polyclonal to Aurora A, rabbit polyclonal to Aurora B, rabbit polyclonal to cleaved caspase 3, rabbit polyclonal to phospho Histone H3 (S10), rabbit polyclonal to p21, rabbit polyclonal to tubulin and rabbit polyclonal to GAPDH were purchased from Cell Signaling (Danvers, MA 01923, USA), mouse monoclonal to Rb protein was purchased from BD Pharmingen (San Jose, CA 95131, USA), mouse monoclonal to p53 from Santa Cruz Biotechnology.

### Quantitative fluorescent microscopy (QFM)

Cells were fixed in 3% paraformaldehyde and the nuclear morphology was examined using fluorescence microscopy after staining with 4′, 6-diamidino-2-phenylindole (DAPI) at a concentration of 25 μg/mL. Number of cells with decondensed, fragmented chromatin was taken as a measure for apoptosis. A minimum of 400 cells were counted for each sample and taken as a percentage of untreated cells.

### Xenograft studies

Athymic mice bearing MPNST or CHP100 tumors (7 mice/cohort) of 150 mm^3^ diameters were either treated with vehicle control, 30 mg/kg of MLN-8237 p.o. once daily 5X for 3 weeks. Twenty-four hours after the treatment on day 12 one animal from each cohort was sacrificed and the tumors examined for H & E, cleaved caspase 3 and Phospho Histone H3 (S10). Tumors were measured every 2 to 3 d with calipers, and tumor volumes were calculated by the formula 4/3 × r3 [r = (larger diameter + smaller diameter)/4]. The percentage of tumor regression was calculated as the percentage ratio of difference between baseline and final tumor volume to the baseline volume. Toxicity was monitored by weight loss. These studies were done in accordance with the Principles of Laboratory Animal Care (NIH Publication No. 85–23, released 1985).

### Time lapse microscopy

HCT116 cells were transiently transfected with green fluorescent protein (GFP)-histone 2B (Addgene, 1 Kendall Sq. Ste. B7102, Cambridge, MA 02139) on 4 well chambered coverglass for 24 hours and treated with either DMSO vehicle or 100 nm or 1000 nm MLN-8237. The chamber was mounted onto the stage of a Zeiss Axiovert 200 M inverted microscope (Carl Zeiss Microimaging, Thornwood, NY) maintaining normal growth condition with Solent Scientific microscope live imaging incubation system (Segensworth, UK). Confocal images of the cells were acquired by a spinning disk confocal system (UltraView, Perkin Elmer, San Jose, CA) with a 20× objective lens (0.8 NA), using a cooled EM-CCD camera (iXon+, Andor Technology, South Windsor, CT) every 5 min after 18 hours of treatment with exposure time limited to 4–5 s/image. For each time point, images were taken with 14 different focal planes along the *z*-axis 6 μm apart. Imaging data were analyzed using MetaMorph (Molecular Devices, Sunnyvale, CA).

### Histopathology

For immunohistochemistry analysis, representative sections were deparaffinized, rehydrated in graded alcohols, and subjected to antigen retrieval by microwave oven treatment using standard procedures. H & E staining was carried out using Gill's hematoxylin (Poly Scientific R & D Corp.) for 10 min as per the manufacturer's protocol followed by counterstaining with eosin (Poly Scientific R & D Corp.) for 4 min. The immunohistochemistry was performed at Molecular Cytology Core Facility of Memorial Sloan Kettering Cancer Center using Discovery XT processor (Ventana Medical Systems). For PHH3S10 (5 mg/mL, Cell Signaling Technologies INC.) and cleaved caspase 3 (Asp175) (0.1 mg/mL, Cell Signaling Technologies INC.) were used followed by biotinylated goat anti-rabbit IgG Streptavidin-HRP and DAB detection kit (Ventana Medical Systems) according to the manufacturer instructions.

### Statistical analysis

All *in vitro* experiments were carried out at least three times. The statistical significance of the experimental results was determined by the two-sided *t* test. We chose *P =* 0.05 as statistically significant in individual comparisons. For *in vivo* studies, the two-sided *t* test was used as a summary measure for each mouse. Tumor volume was compared between groups of mice. *P* values were calculated using the Wilcoxon Rank Sum test.

## SUPPLEMENTARY MOVIES




